# Association Between Eating Disorders and Type 1 Diabetes Mellitus: a Systematic Review and Meta-Analysis

**DOI:** 10.1192/j.eurpsy.2024.487

**Published:** 2024-08-27

**Authors:** Y. E. Dean, K. R. Motawea, M. Aslam, J. J. Loayza Pintado, H. A. Popoola-Samuel, M. Salam, P. O. R. Dundi, W. Donaldy, E. M. AlEdani, Z. AlQiqie, N. Sultana, A. R. H. Mohamed, A. Elalem, S. T. H. Syeda, M. S. Mohamed, M. W. Assal, N. M. Attia, H. Hagar, H. A. Abdelaziz, M. L. P. Le, A. Elbahaie, Y. Hazimeh, H. Aiash

**Affiliations:** ^1^Alexandria University, Faculty of Medicine, Alexandria, Egypt; ^2^Shaikh Khalifa Bin Zayed Al-Nahyan Medical and Dental College, Lahore, Pakistan; ^3^Universidad de San Martin de Porres Facultad de Medicina Humana, Lima, Peru; ^4^College of Health and Sciences, Rush University, Chicago, United States; ^5^Mediclinic City Hospital, Dubai, United Arab Emirates; ^6^Karnataka Institute of Medical Sciences, RHUHS, Hubli, India; ^7^Harlem Hospital Center, New York City, United States; ^8^University of Basra, Medical College, Basra, Iraq; ^9^Odessa national medical university, Odessa, Ukraine; ^10^Shadan Institute of Medical Sciences, Hyderabad, India; ^11^Faculty of Medicine, Suez Canal University, Ismailia, Egypt; ^12^Deccan College of Medical Sciences, Telangana, India; ^13^Zagazig University, Faculty of Medicine, Zagazig; ^14^Alexandria University, High Institute of Public Health, Alexandria, Egypt; ^15^Poznan University of Medical Sciences, Ponzan, Poland; ^16^Ibn Sina National College, Jeddah, Saudi Arabia; ^17^Lebanese University, Beirut, Lebanon; ^18^SUNY Upstate Medical University, Syracuse, United States

## Abstract

**Introduction:**

Type 1 diabetes mellitus (T1DM) patients are treated via insulin which could result in weight gain. Studies have coined a new term, “Diabulimia” which refers to the limitation or skipping of insulin doses, with the objective of weight control. A previous meta-analysis has found that eating disorders (ED) are significantly associated with T1DM (Mannucci, E et al. J Endocrinol Invest 2005; 417-9), while a more recent one, has shown an insignificant association between ED and T1DM on analysis of diabetes-adapted questionnaires only (Young V, et al. Diabet Med. 2013:189-198)

**Objectives:**

We aimed to re-analyze the association between ED and T1DM, whilst taking into account recently published literature and the type of questionnaire utilized.

**Methods:**

A literature search of PubMed, Scopus, and Web of Science was conducted on 17th January 2023, using the key terms “ T1DM”, “Eating Disorders”, and “ Bulimia”. Only Observational controlled studies were included.

**Results:**

T1DM was associated with increased risk of ED compared to non-diabetic individuals (RR = 2.47, 95% CI = 1.84 to 3.32, p-value < 0.00001), especially bulimia nervosa (RR = 2.80, 95% CI = 1.18 to 6.65, p-value = 0.02) and binge eating (RR = 1.53, 95% CI = 1.18 to 1.98, p-value = 0.001), while no significant association was seen between T1DM and anorexia nervosa. Our sensitivity analysis has shown that increased risk of ED among T1DM persisted regardless of the questionnaire used to diagnose ED; DM-validated questionnaires (RR = 2.80, 95% CI = 1.91 to 4.12, p-value <0.00001) and generic questionnaires (RR = 2.03, 95% CI = 1.27 to 3.23, p-value = 0.003). Furthermore, the Eating Attitudes Test-26 (EAT) showed a significant increase in the dieting subscale (MD = 2.95, 95% CI = 1.84 to 4.06, p-value < 0.00001) and bulimia subscale (MD = 0.78, 95% CI = 0.12 to 1.44, p-value = 0.02) among T1DM patients. Additionally, the Bulimic Investigatory Test, Edinburg (BITE) showed a significant increase in the symptom subscale (MD = 0.31, 95% CI = 0.12 to 0.50, p-value = 0.001), however, no significant difference was detected between T1DM and controls in the severity subscale. Prevalence of insulin omission/misuse was 10.3% (95% CI = 8.1-13); diabetic females demonstrated significantly higher risk of insulin omission (RR = 14.21, 95% CI = 2.66 to 76.04, p-value = 0.002) and insulin misuse (RR = 6.51, 95% CI = 1.14 to 37.31, p-value = 0.04) compared with diabetic males. Analysis of other potentially unhealthy weight control behaviors showed insignificant associations between fasting, excessive exercise, dieting pills misuse, diuretics misuse, and T1DM.

**Conclusions:**

T1DM patients are at higher risk of developing ED according to both generic and diabetes-validated questionnaires. Moreover, female diabetics are at higher risk of insulin misuse/omission. Subsequently, patients should be regularly screened and early psychiatric management is warranted.

**Disclosure of Interest:**

None Declared

